# GO-Tibia: a masked, randomized control trial evaluating gentamicin versus saline in open tibia fractures

**DOI:** 10.1186/s13063-023-07410-0

**Published:** 2023-06-15

**Authors:** Billy T. Haonga, Jamieson M. O’Marr, Patrick Ngunyale, Joshua Ngahyoma, Justin Kessey, Ibrahim Sasillo, Patricia Rodarte, Tigist Belaye, Eleni Berhaneselase, Edmund Eliezer, Travis C. Porco, Saam Morshed, David W. Shearer

**Affiliations:** 1 Muhimbili Orthopaedic Institute, Muhimbili University of Health and Allied Sciences, Dar Es Salaam, Tanzania; 2 Department of Orthopaedic Surgery, Institute for Global Orthopaedics and Traumatology, University of California San Francisco School of Medicine, 2550 23rd Street, Building 9, 2nd Floor, San Francisco, CA 94110 USA; 3 F.I. Proctor Foundation, University of California San Francisco, 513 Parnassus Avenue, San Francisco, CA 94122 USA; 4 Department of Ophthalmology, University of California San Francisco, 10 Koret Way, San Francisco, CA 94143 USA; 5 Department of Epidemiology and Biostatistics, University of California San Francisco, 550 16th St. 2nd Floor, San Francisco, CA 94158 USA

**Keywords:** Local antibiotics, Fracture-related Infection, Open tibia fractures, Gentamicin, Tanzania, Randomized control

## Abstract

**Background:**

The rate of open tibia fractures is rapidly increasing across the globe due to a recent rise in road traffic accidents, predominantly in low- and low-middle-income countries. These injuries are orthopedic emergencies associated with infection rates as high as 40% despite the use of systemic antibiotics and surgical debridement. The use of local antibiotics has shown some promise in reducing the burden of infection in these injuries due to increasing local tissue availability; however, no trial has yet been appropriately powered to evaluate for definitive evidence and the majority of current studies have taken place in a high-resource countries where resources and the bio-burden may be different.

**Methods:**

This is a prospective randomized, masked, placebo-controlled superiority trial designed to evaluate the efficacy of locally administered gentamicin versus placebo in the prevention of fracture-related infection in adults (age > 18 years) with primarily closeable Gustillo-Anderson class I, II, and IIIA open tibia fractures. Eight hundred ninety patients will be randomized to receive an injection of either gentamicin (treatment group) or saline (control group) at the site of their primarily closed open fracture. The primary outcome will be the occurrence of a fracture-related infection occurring during the course of the 12-month follow-up.

**Discussion:**

This study will definitively assess the effectiveness of local gentamicin for the prevention of fracture-related infections in adults with open tibia fractures in Tanzania. The results of this study have the potential to demonstrate a low-cost, widely available intervention for the reduction of infection in open tibia fractures.

**Trial registration:**

Clinicaltrials.gov NCT05157126. Registered on December 14, 2021.

## Administrative information

Note: The numbers in curly brackets in this protocol refer to the SPIRIT checklist item numbers. The order of the items has been modified to group similar items (see http://www.equator-network.org/reporting-guidelines/spirit-2013-statement-defining-standard-protocol-items-for-clinical-trials/).

**Table:** 

Title {1}	GO-Tibia: a masked, randomized control trial evaluating Gentamicin versus saline in open tibia fractures
Trial registration {2a and 2b}	Clinicaltrials.gov identifier: NCT05157126
Protocol version {3}	Version 3.0, Date: 1/27/22
Funding {4}	Funding was received from: National Institute of Arthritis and Musculoskeletal and Skin Diseases (NIAMS), USA Orthopedic Research and Education Foundation, USA
Author details {5a}	Billy T Haonga, Muhimbili Orthopaedic Institute, Muhimbili University of Health and Allied Sciences, Dar es Salaam, Tanzania. Jamieson M. O’Marr, Institute for Global Orthopaedics and Traumatology, Department of Orthopaedics, University of California, San Francisco, San Francisco, CA Patrick Ngunyale, Muhimbili Orthopaedic Institute, Dar es Salaam, Tanzania. Joshua Ngahyoma, Muhimbili Orthopaedic Institute, Dar es Salaam, Tanzania. Justin Kessey, Muhimbili Orthopaedic Institute, Dar es Salaam, Tanzania. Ibrahim Sasillo, Muhimbili Orthopaedic Institute, Dar es Salaam, Tanzania.
	Patricia Rodarte, Institute for Global Orthopaedics and Traumatology, Department of Orthopaedics, University of California, San Francisco, San Francisco, CA Tigist Belaye, Institute for Global Orthopaedics and Traumatology, Department of Orthopaedics, University of California, San Francisco, San Francisco, CA Eleni Berhaneselase, Institute for Global Orthopaedics and Traumatology, Department of Orthopaedics, University of California, San Francisco, San Francisco, CA Edmund Eliezer, Muhimbili Orthopaedic Institute, Dar es Salaam, Tanzania. Travis C. Porco, Department of Epidemiology and Biostatistics, University of California San Francisco, San Francisco, CA Saam Morshed, Institute for Global Orthopaedics and Traumatology, Department of Orthopaedics, University of California, San Francisco, San Francisco, CA David W. Shearer, Institute for Global Orthopaedics and Traumatology, Department of Orthopaedics, University of California, San Francisco, San Francisco, CA
Name and contact information for the trial sponsor {5b}	Dr. David Shearer Institute for Global Orthopaedics and Traumatology, University of California, San Francisco, San Francisco, CA 2550 23rd Street, Building 9, 2nd Floor San Francisco, CA, 94,110 David.shearer@ucsf.edu Tel: (628) 206–8812
Role of sponsor {5c}	The sponsor is involved in the study design; collection, management, analysis, and interpretation of the data; writing of the report; and the decision to submit the report for publication. Funders are not involved in any of the aforementioned activities.

## Introduction

### Background and rationale {6a}

Open tibia fractures are orthopedic emergencies associated with a very high rate of morbidity and complications. These injuries can have infection rates of up to 40% and are at a high risk of other complications such as severe soft tissue damage, nonunion, and amputation [1, 2]. The rate of open tibia fractures continues to rise globally, largely due to increases in road traffic accidents, representing a substantial amount of orthopedic morbidity [3]. This is particularly true in low- and middle-income countries where the rates of road traffic accidents continue to increase at alarming rates.

The standard of care treatment for open tibia fractures includes debridement, irrigation, soft-tissue coverage, osseus stabilization, and systemic antibiotic prophylaxis, yet despite these measures, complications remain high [4, 5]. The high rates of infection observed are in part due to soft tissue injury that compromise the blood supply to the affected region and thus limit the availability of the systemic antibiotic prophylaxis [6]. Local antibiotics have been proposed as an additional treatment to increase local bioavailability and potentially reduce infection rates [7, 8].

Previous research regarding the use of local antibiotics for the prevention of fracture-related infections (FRI) in open tibia fractures has demonstrated some positive results. A systematic review including data from nearly 3000 patients demonstrated a reduction in FRI of nearly 12% with the use of local antibiotics in various forms [9]. More recently, an open-label clinical trial conducted in the USA demonstrated a significant reduction in gram-positive organisms with the use of local vancomycin [10]. Despite these positive results, a properly powered trial for definitive evidence remains to be performed. Additionally, most of the current open tibia fracture research involving local antibiotics has taken place in high-income countries where the bioburden and healthcare resources can be substantially different from low- and middle-income countries [11, 12].

In this study, we aim to assess the effectiveness of local gentamicin for the prevention of FRI in open tibia fractures in Tanzania. We will initiate randomized, masked, placebo-controlled trial at a single tertiary care center in Dar es Salaam, Tanzania, to assess whether local gentamicin injection is superior compared to a saline placebo injection.

### Objectives {7}

The primary objective of this study is to determine the efficacy of intraoperative, locally administered gentamicin versus placebo in the management of open tibial shaft fractures as measured by the prevention of fracture-related infection (FRI). Secondary objectives include assessing the efficacy of local gentamicin versus placebo on the occurrence of nonunion and unplanned fracture-related reoperations.

### Trial design {8}

This is a randomized, masked, placebo-controlled superiority trial to evaluate the efficacy of locally administered gentamicin versus placebo in the prevention of fracture-related infection in open tibia fracture patients. Patients will be randomized using randomly permuted blocks in a 1:1 allocation ratio.

## Methods: participants, interventions, and outcomes

### Study setting {9}

The study will take place at Muhimbili Orthopaedic Institute (MOI), a national academic hospital in Dar es Salaam, Tanzania.

### Eligibility criteria {10}

A patient must meet all of the following inclusion criteria to be eligible for inclusion:

1. Skeletally mature (age > 18 years old)
2. Possess an open tibial shaft fracture meeting the following criteria:
  - OTA type 42
  - Primarily closable wound
  - Gustilo-Anderson classes I, II, and IIIA

A potential patient that meets any of the following criteria will be excluded from participation in the study:

1. Time from injury to presentation at the Muhimbili Orthopaedic Institute greater than 48 h
2. Time from injury to surgery greater than 7 days
3. Aminoglycoside allergy
4. Gustilo-Anderson class IIIB or IIIC
5. Bilateral open tibial fractures
6. Severe brain injury (GCS < 12)
7. Spinal cord injury
8. Severe vascular injury
9. Severe burns (with greater than 10% total body surface injury or greater than 5% of total body surface area with full thickness or circumferential injury)
10. Pathologic fracture
11. History of ipsilateral, active limb infection
12. End-stage renal disease on hemodialysis
13. Patient is unlikely to complete the full year of follow-up

If the patient meets all the inclusion criteria and does not meet any exclusion criteria, the patient will be considered eligible for the study.

### Who will take informed consent? {26a}

The study protocol will be introduced to eligible patients by the research coordinator on duty at the time of their presentation to the MOI emergency department. If interested in participating, the research coordinator will explain the full details and expectations of participation in the study. Patients will then conduct the informed consent with the research coordinator, and both participants will sign and date the informed consent document which will be preserved in a secure location at the study site. Should a patient be unable to sign a written consent, a mark of approval will be made by the participant in the participant signature section and a witness to the informed consent process will sign below indicating they observed the process and answered any additional patient questions alongside the research coordinator.

### Additional consent provisions for collection and use of participant data and biological specimens {26b}

No additional consent provisions are included, and no biological specimens will be collected as part of this study.

### Interventions

#### Explanation for the choice of comparators {6b}

A matched placebo of normal saline will be used as the comparator group. Saline provides an inert sterile solution that visually matches the study solution that contains the dose of gentamicin, allowing for the masking of the study intervention from the care providers at the time of administration. All other standard of care measures in the treatment of open tibial fractures will be offered to all participants.

#### Intervention description {11a}

A certified unmasked study nurse will prepare 5-ml aqueous gentamicin (16 mg/ml) and 5-ml normal saline solutions. The solutions are prepared in identical syringes labeled with the solution expiration date and either “solution A” or “solution B” according to the masking key before they are stored in a locked study fridge adjacent to the operating theater. The masking key is only accessible to the study nurse. Solutions are stored at 4 °C for up to 48 h, per pharmacist guidelines, and a solution preparation log is maintained to ensure the integrity of the study solutions.

**Fig. 1:**
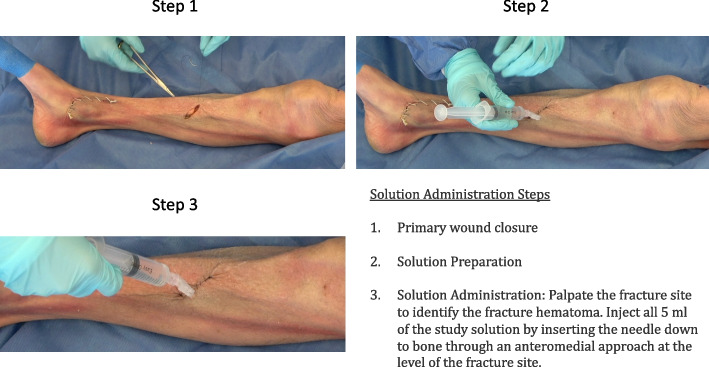
Example of injection administration. Step 1: Assess ability to close wound primarily. If able, proceed with watertight closure. Step 2: Palpate the fracture site with the needle to ensure study solution enters the fracture hematoma. Step 3: Inject full study solution

At the conclusion of the index surgical procedure (wound debridement, skeletal stabilization, as appropriate), the surgeon confirms that the wound can be closed primarily. At that point, the patient is randomized using the Research Electronic Data Capture (REDCap) randomization tool. The appropriate study solution is then injected into the closed wound using a 22-gauge needle down to the bone through an anteromedial approach at the level of the fracture site using a sterile technique (Fig. 1).

#### Criteria for discontinuing or modifying allocated interventions {11b}

N/A: Because the intervention is a single injection administered intraoperatively under anesthesia, withdrawal or modification of the intervention after administration is not applicable. Patients may withdraw from the study follow-up procedures at any time.

#### Strategies to improve adherence to interventions {11c}

N/A: The study includes only a single intervention at the time of enrollment and thus adherence to the intervention is not applicable.

#### Relevant concomitant care permitted or prohibited during the trial {11d}

All patients will undergo standard of care treatment for open tibial shaft fractures at MOI regardless of their eligibility for enrollment in the study. This includes several perioperative co-interventions:

- Systemic antibiotic prophylaxis: All patients will receive a single dose of 1-g IV ceftriaxone as soon as possible after presentation to the hospital.
- Surgical debridement: All patients will undergo systematic debridement of the traumatic wound with the removal of any devitalized bone and soft tissue. Once the surgeon has deemed appropriate debridement has taken place the wound will be irrigated.
- Fracture stabilization: Fixation of the fracture will be at the discretion of the treating surgeon. It will be temporizing or definitive using either external fixation (EF) or intramedullary nailing (IMN). External fixation will be with a uniplanar external fixator consisting of a minimum of two Schanz pins proximal to the fracture and two Schanz pins distal to the fracture connected by a single stainless steel bar. Pin care protocol will consist of cleaning the pin sites with methyl alcohol twice daily for the duration of the external fixator placement. Intramedullary nailing will utilize the SIGN intramedullary nail system. An infrapatellar approach will be utilized with two proximal and two distal locking screws placed using an external jig. Intraoperative fluoroscopy will not be used routinely.
- Wound closure and wound care: The wound will be closed primarily when it is felt safe and feasible to do so by the operating surgeon. The wound will be checked at the 2-week visit, and if dry the dressing will be removed. If it is not, the patient will be instructed to perform additional wound care.
- Weight bearing protocol: All patients will be advised to be on toe-touch status for the first 6 weeks following surgery. Afterwards, the patient will be instructed to weight bear as permitted by pain.

The study has no prohibited concomitant care. Patients may continue to use any additional medications so long as it is not placed locally around the wound site. If a medication is applied locally to the wound site, it will be recorded as a protocol violation.

#### Provisions for post-trial care {30}

All participants will receive postoperative care and treatment for adverse events according to the local standard of care. The study does not cover the cost of care for adverse events but follow-up visits and imaging will be free for all participants.

### Outcomes {12}

#### Primary outcomes

The primary outcome of this study will be the occurrence of fracture-related infection (FRI), a binary outcome. FRI is a consensus definition of infection after fracture treatment that is clinically diagnosed by an orthopedic surgeon based on any of the following four diagnostic criteria: (1) fistula, sinus, or wound breakdown; (2) purulent drainage from the wound or presence of pus during surgery; (3) phenotypically indistinguishable pathogens identified by culture from at least two separate deep tissue/implant specimens; or (4) presence of microorganisms in deep tissue taken during an operative intervention, as seen on histopathological examination. It is expected that the peak diagnosis time will occur between 6 weeks and 6 months; however, these criteria will be assessed by an orthopedic surgeon at every follow-up time point.

#### Secondary outcomes

The following are the secondary outcomes:

1. Compare the rates of nonunion, a binary variable between the two treatment groups at 12 months after the initial treatment. Nonunion is defined as follows:
  - Any unplanned reoperation for the promotion of bone healing
  - mRUST ≤ 10 AND either FIX-IT score ≤ 11- at 12-month follow-up OR recommendation by treating surgeon for nonunion repair surgery
2. Occurrence of unplanned fracture-related reoperation, a binary variable, for infection, wound healing, or fracture union, excluding the removal of implants for prominence/irritation within 12 months of injury. This may include but is not limited to the following:
  - Irrigation and debridement of surgical incisions or open fracture wounds due to infections or wound healing problems
  - Revision wound closure for dehiscence
  - Soft tissue coverage procedure for infected or necrotic wound
  - Fracture-delayed union or nonunion surgery (such as bone grafting or implant exchange)
  - Reoperation for hardware or prosthesis failure due to infection or bone-healing problems
  - Amputation for infection, wound, or fracture healing problem
3. Compare the two intervention groups for differences in radiographic healing using the modified RUST scores at each follow-up time point with appropriate radiographs (3, 6, 9, and 12 months)
4. Compare the two intervention groups for differences in clinical union outcomes at each follow-up time point using the FIX-IT score (3, 6, 9, and 12 months)
5. Compare the two intervention groups for differences in health-related quality of life at 12 months after fracture treatment using the EuorQol EQ-5D survey
6. Calculate the cost-effectiveness of the intervention through the quantification of the direct costs, indirect costs, and utility scores for open tibia fractures over the 12-month period following injury

### Participant timeline {13}

**Fig. 2:**
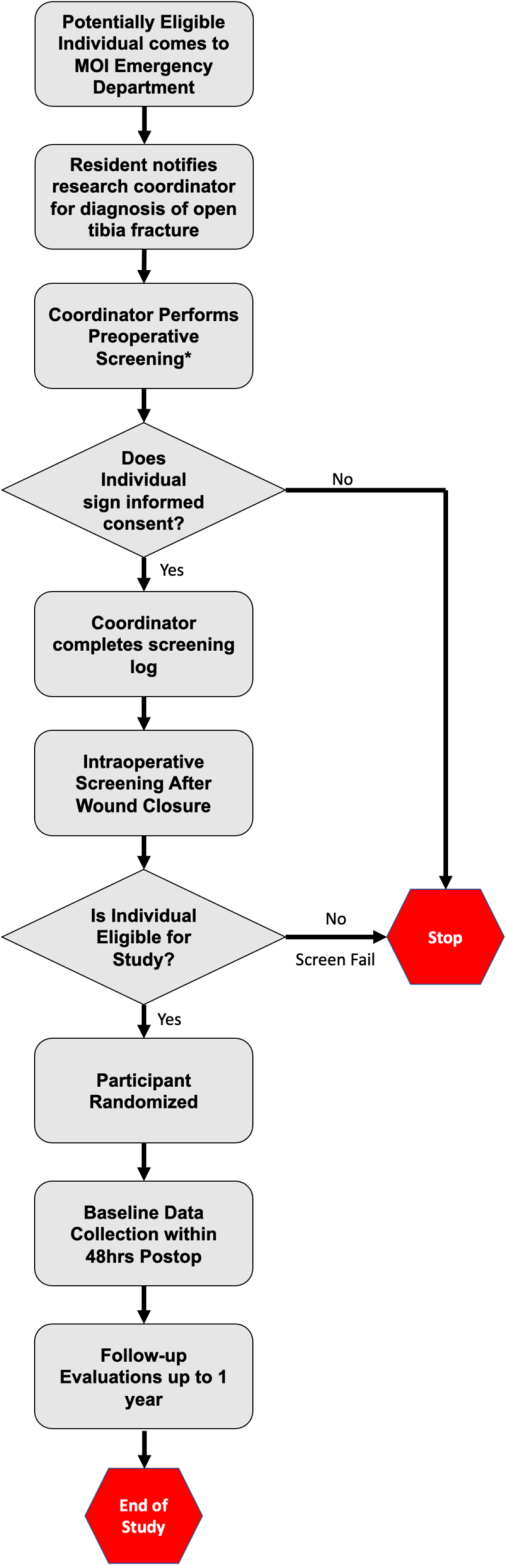
Study flow diagram

The process for screening and enrollment can be seen in the study flow diagram shown in Fig. 2. The schedule of baseline data collection and follow-up assessments is shown in Table 1. After informed consent, the participant is taken to the operating room for debridement and skeletal stabilization. After wound closure while under anesthesia, the randomly allocated intervention is administered. Baseline clinical and demographic data are collected within 48 h of the intervention prior to discharge from the hospital.

**Table 1:** GO-Tibia schedule of events

Assessment	Hospital				Outpatient					
	Pre-surgery		Surgery	Post- surgery, ≤ 48 h postop	2 weeks	6 weeks	3 months	6 months	9 months	12 months
	Screen	Enroll								
Radiographs	●			●			●	●	●	●
Informed consent		●								
Serum creatinine		●		●						
Randomization			●							
Intervention			●							
Baseline data				●						
Contact information				●						
EQ-5D				●			●	●	●	●
Outcomes										
Assessment					●	●	●	●	●	●
FIX-IT							●	●	●	●
WPAI						●	●	●	●	●
C-reactive protein					●	●	●	●	●	●
Adverse event										
Screen					●	●	●	●	●	●

Post-discharge follow-up assessments are performed at 2 weeks, 6 weeks, 3 months, 6 months, 9 months, and 12 months after the intervention.

### Sample size {14}

Data from a prior randomized control trial conducted at Open Tibia Trial (MOI) and the FLOW trial was used to estimate the base rate of reoperation at 12% [13, 14]. Previous literature suggests using local aminoglycoside antibiotics provides a relative risk reduction of approximately 50% for FRI [15]. An absolute risk reduction of 12 to 6% with a two-tailed alpha of 0.05, a power of 80%, and an estimated loss to follow-up rate of 20% suggests a sample size of 445 individuals per treatment arm for a total of 890 enrolled patients.

### Recruitment {15}

When a patient presents with an open tibial fracture that is potentially eligible for inclusion in the study, the orthopedic provider in the emergency department contacts the research coordinator on-call. To avoid missing potentially eligible patients, the research coordinators regularly review the emergency department admissions records twice daily for patients with a diagnosis of open tibial fracture.

Upon notification of a potentially eligible patient, pre-screening is performed by coordinators using the medical record and imaging already obtained as part of routine medical care. If eligible based on pre-screening, patients are invited to participate in the study and informed consent is provided. After obtaining consent, the final screening is performed, which includes an intraoperative assessment that the wound is amenable to primary closure.

### Assignment of interventions: allocation

#### Sequence generation {16a}

The randomization seed will be generated using R. The script randomization.Rnw will be used to conduct the random shuffle with permuted blocks of equal frequency. Note that the choice of the random number seed completely determines the randomization.

#### Concealment mechanism {16b}

The allocation sequence generated by the statistician in R will be distributed to a REDCap server, which is accessed at the point of randomization by the study coordinators after confirmation of primary skin closure in the operating room. The investigators and field teams are strictly masked with respect to the allocation sequence. A secure password-protected copy of the sequence will be maintained at UCSF accessible only by two unmasked research staff members.

#### Implementation {16c}

The randomization sequence will be generated by the study statistician, but the statistician will not have access to the masking key and hence will not know whether solution A or B is the intervention or placebo. The research coordinators at the study site are responsible for enrolling participants and assigning the participant to receive solution A or B using the REDCap randomization module.

### Assignment of interventions: blinding

#### Who will be blinded {17a}

Trial participants, care providers, research coordinators involved in data collection and monitoring, outcome assessors, and the study statisticians are masked. The only members of the study team with access to the masking key are the two study nurses that prepare the active and placebo solutions and two senior research staff members at the coordinator center. To assess for unmasking, the surgeons are asked to guess whether the solution is active or placebo at the time of administration, and these data are monitored at DSMC meetings.

#### Procedure for unblinding if needed {17b}

The data safety and monitoring committee and NIH-appointed safety officer have the option to be unmasked at any point if there is a concern for participant safety.

### Data collection and management

#### Plans for assessment and collection of outcomes {18a}

All data collection will be carried out by the 3 research coordinators. The research coordinators have undergone training on the proper employment of all appropriate scales and surveys used in the study.

All patients possessing potentially eligible open tibia fractures will be screened by the program coordinators. Should a patient prove eligible and provide informed consent, basic demographic information, including socioeconomic status, medical and social history, injury characteristics, and estimated pre-injury quality of life, will be collected. Health-related quality of life will be measured using the validated and widely used EuroQol EQ-5D survey [16]. Personal contact information will be obtained along with contact information for at least two close contacts to optimize follow-up. Preoperative orthogonal radiographs and serum creatinine will also be obtained.

The patient will then proceed for operative treatment and definitive fixation at the discretion of the treating surgeon. Wound size measurements (using a centimeter ruler or the handle of a scalpel) will be taken of the maximum dimension of the wound both prior to debridement and after debridement. OTA open fracture classification, Gustillo-Anderson classification, fracture fixation data, and the total time to fixation will be recorded.

Following the intervention, postoperative orthogonal radiographs and serum creatine collected on postoperative day 2 will be obtained. Preoperative radiographs will undergo OTA fracture classification and postoperative radiographs will be measured for any coronal or sagittal angulation by a trained member of the coordinating center. Prior to discharge, the research coordinator will meet with each patient to schedule their follow-up appointments and document any in-hospital complications.

At the 2-week follow-up appointment, serum creatinine and CRP will be drawn. Patients will complete the outcomes assessment surveys for fracture-related infection, current pain level, unplanned fracture-related reoperation, and other adverse events [17]. At the 6-week follow-up, only the CRP will be drawn and the patient will again complete all the same surveys done at the 2-week visit.

For all remaining study visits at 3, 6, 9, and 12 months, patients will complete radiographs of the affected limb and have a CRP drawn. Radiographs will be evaluated for their degree of coronal or sagittal angulation and the modified RUST score will be completed by a member of the coordinating center [18]. Patients will then complete all prior described surveys including fracture-related infection, current pain level, unplanned fracture-related reoperation, and adverse events. In addition, the patient will complete the EQ-5D survey and undergo a clinical examination by an attending orthopedic surgeon for the completion of the FIX-IT score [19].

#### Plans to promote participant retention and complete follow-up {18b}

All attempts will be made to encourage patients to adhere to the study follow-up schedule. Contact information for the patient and two close contacts are obtained to maximize our chances of maintaining contact. Each patient will be reminded by phone the week preceding their follow-up appointment. To ease the burden of follow-up, all study patients will also be seen for follow-up in a dedicated study clinic and will have the fees for their radiographs, follow-up, and necessary bloodwork covered by the study. In the event a patient is unable to make a follow-up appointment in their dedicated follow-up window, a telephone fracture-related infection screen will be completed and attempts to reschedule future appointments will be made.

Patients are made aware during the consenting process that enrollment in the study that patients will incur the standard fees for evaluation and treatment; however, to decrease the financial burden of study participation and follow-up, the following procedures will be covered for all participants:

- Systemic antibiotics for all screened patients
- Preoperative, postoperative, and 2-week creatine levels
- C-reactive protein levels at all study follow-ups
- All follow-up radiographs
- Follow-up consolation fees for the follow-up clinical evaluations at a dedicated study clinic to minimize wait times
- Two intraoperative cultures for any study patient who undergoes a reoperation

### Data management {19}

Three trained research coordinators will be responsible for all data collection, working in conjunction with providers at the study site when required. All data will be entered and stored on REDCap, a secure data management platform only accessible to members of the study, using portable laptops and phones. The REDCap system uses a variety of mechanisms to ensure data quality including skip logic, range checks, and data type checks. A member of the coordinating center will do weekly checks on the entered data for the duration of the study to ensure completeness. The full range of data quality and monitoring can be found in the trial DSMP.

### Confidentiality {27}

All data will be maintained on REDCap, a secure data management tool that requires dual authentication and is only accessible to those approved to work with study data. In addition, all participants are assigned a unique identifier. This identifier is used for all data monitoring and analysis. Patient identifying information is only available to the local study coordinators and one member of the coordinating center.

### Plans for collection, laboratory evaluation, and storage of biological specimens for genetic or molecular analysis in this trial/future use {33}

The standard laboratory protocols will be used at MOI for the collection and testing of blood specimens for all study-related tests including serum creatinine and C-reactive protein. Should a study participant undergo reoperation, cultures will be collected and performed at the Muhimbili University of Health and Allied Sciences (MUHAS) laboratory. No biological specimens will be stored for genetic or molecular analysis in the future.

## Statistical methods

### Statistical methods for primary and secondary outcomes {20a}

#### Statistical methods for primary outcome

The primary analysis will surround the occurrence of FRI as a binary outcome. This will be done through a binomial regression with the complementary log–log link, allowing for the available person-time to be used. The estimated effect will be the relative hazard. The analysis will be two-sided, with a type I error rate (alpha) of 0.05. Each trial is separate, with an independent alpha.

### Statistical methods used for secondary and additional outcomes

These analyses are designed to provide additional insight and support for the primary analysis and to assess whether the methodological choices we made had an undue effect on the results. In reporting, they will be sharply distinguished from the primary outcome. Additional sensitivity analyses will be conducted based on the following variables as baseline covariates: Gustilo-Anderson fracture type, OTA Open Fracture Classification for contamination, time from injury to first debridement, and method of fixation. We will make available the results of Fisher’s exact test on the 2 × 2 cross-tabulation of the reoperation binary outcome and the treatment assignment. This does not consider the observation time and thus is an independent statistical procedure and may yield different decisions than the primary prespecified analysis. We will also report the 95% confidence interval for the risk difference between the groups. Because this will be conducted by an independent regression (binomial regression with the identity link), these new findings may be statistically inconsistent with the main analysis. Thus, we will only report 95% confidence intervals, omitting *P*-values.

The risk difference is interpretively important and may be useful in future meta-analytic studies. Intent-to-treat analysis is recommended but impossible when the primary outcome is missing. We will conduct exploratory regression of missingness and observation time, using the following predictors when appropriate: gender, severity of injury, cost of travel to clinic, and age.

### Interim analyses {21b}

Interim analysis will be conducted by the trial statistician and reported to the DSMC when the milestones of 1/3 target enrollment and 2/3 of target enrollment have been met. Statistical evidence of harm at either interim analysis will result in trial discontinuation. In addition, both interm analysis events will evaluate futility using a cutoff of a conditional power to detect 20% as the threshold for either discontinuation of the trial or protocol changes at the discretion of the DSMC.

### Methods for additional analyses (e.g., subgroup analyses) {20b}

We will evaluate the several subgroups of clinical interest for any differential treatment effects of local gentamicin administration. These groups include (i) Gustillo-Anderson classification, (ii) severity of wound contamination (minimal vs moderate vs severe using the OTA open fracture classification for contamination), (iii) time from injury to surgery (< 24 h vs > 24 h), and (iv) type of definitive fixation (external fixation vs intramedullary nailing). This analysis will be reported descriptively and with confidence intervals, but no significance *P*-values will be given. Conclusions based on such subgroups or adjustments will be considered hypothesis-generating and will not form the basis for highlighting in reports or publications.

### Methods in analysis to handle protocol non-adherence and any statistical methods to handle missing data {20c}

Intent-to-treat analysis will be performed where possible. Missing data in any study outcome will not undergo imputation. We will conduct exploratory regression of missingness and observation time, using the following predictors when appropriate: gender, severity of injury, cost of travel to clinic, and age.

### Plans to give access to the full protocol, participant-level data, and statistical code {31c}

Non-identifiable patient-level data and analytical code may be made available upon reasonable request.

### Oversight and monitoring

#### Composition of the coordinating center and trial steering committee {5d}

The coordinating center is the University of California, San Francisco. There are weekly meetings between the coordinating center principal investigator, project managers, and research fellows where any data quality or study-related issues are discussed. In addition, there are bi-monthly meetings with the study site principal investigator and research coordinators where study progress and issues are discussed. In addition, the trial is overseen by the trial steering committee, consisting of the statistician and two orthopedic surgeons from the coordinating center. The adjudication committee consisting of two orthopedic surgeons from the coordinating center and one Tanzanian orthopedic surgeon meet every 2 months to adjudicate any suspected cases of the primary outcome.

**Fig. 3:**
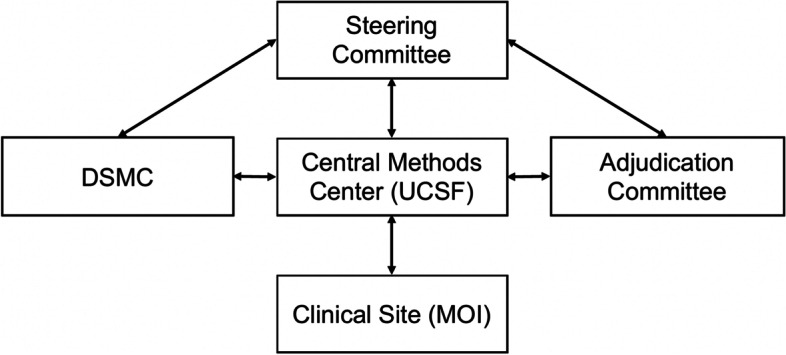
Study organization chart

Finally, the trial has additional external oversight by both the NIAMS-appointed monitoring body and the DSMC. An organization chart of all involved organizations can be seen in Fig. 3.

#### Composition of the data monitoring committee, its role, and reporting structure {21a}

The DSMC will provide safety and efficacy monitoring for the duration of the study. The DSMC consists of a group of multidisciplinary experts with experience in trial management and consists of a US-based orthopedic surgeon, a Tanzania-based emergency medicine physician, a PhD health services researcher, and a NIAMS-appointed safety officer. All DSMC members are independent of the study sponsors and both the coordinating and study site.

The DSMC will provide recommendations on continuing or stopping of the trial. In addition, they may provide recommendations on recruitment, follow-up, and/or data management during the duration of the trial. The DSMC will have the power to prematurely stop the trial for considerations of safety or futility during their regular reviews of serious adverse events or interim analysis.

#### Adverse event reporting and harms {22}

All severe adverse events (SAEs) will be immediately reviewed by the steering committee and reported to the UCSF IRB, Tanzanian IRB, and the NIAMS-appointed safety officer. Serious adverse events consist of any of the following:

- Results in death
- Is life-threatening
- Requires inpatient hospitalization or prolongation of existing hospitalization
- Results in a persistent or significant disability/incapacity
- Results in congenital anomaly/birth defect
- Any other adverse event that, based upon appropriate medical judgment, may jeopardize the subject’s health and may require medical or surgical intervention to prevent one of the other outcomes listed in this definition

Adverse events (AEs) will be screened for at every follow-up visit using the question “Have you developed any new health problems since the previous visit?”. AEs will be recorded on a separate form documenting the date of onset and recovery, seriousness/severity, and outcome. Each AE will be classified as either expected/unexpected and definitely related/possibly related/definitely not related by the DSMC. AEs will be reviewed every 6 months during standard DSMC meetings.

#### Frequency and plans for auditing trial conduct {23}

Due to the quality of medical records at the Tanzanian site, there is no plan for formal auditing. However, site visits will be performed twice per year to observe and ensure compliance with study procedures.

#### Plans for communicating important protocol amendments to relevant parties (e.g., trial participants, ethical committees) {25}

Protocol amendments which may affect the conduct of the study or the potential safety or benefits to participants will require a formal amendment to the protocol. This formal amendment will be approved by the steering committee and must be approved by the UCSF IRB, Tanzanian IRB, and NIAMS. Administrative changes (minor corrections or clarifications that have no effect on the conduct of the trial) will not need to undergo a formal amendment process. These minor changes will be recorded by the coordinating center.

#### Dissemination plans {31a}

Results from the study will be submitted for publication in relevant international peer-reviewed journals by the study principal investigators.

## Discussion

In this study, we aim to definitively assess the effectiveness of local gentamicin for the prevention of fracture-related infections in open tibia fractures in Tanzania. After a successful feasibility trial, we have initiated an appropriately powered randomized clinical trial [20, 21]. To our knowledge, this represents the first clinical trial to evaluate locally applied gentamicin for open tibial fractures in a low-income country.

The strengths of this study include random allocation of the intervention and placebo control with masking of participants, care providers, research coordinators, outcome assessors, and the study statistician. The study results have the potential to reduce morbidity from infection after open tibial fracture using a low-cost, widely available intervention. Furthermore, results are likely to be generalizable not only to low-resource settings, but high-income countries as well.

Conducting a robust clinical trial in low-income countries can pose unique operational and ethical challenges. We have previously demonstrated through both a pilot trial and previous research studies that these challenges can be largely addressed and overcome through academic partnering [13, 22, 23]. There are also important ethical considerations when conducting a clinical trial in a region where there is potential for exploitation of economically disadvantaged populations. In this trial, the study intervention is a low-cost medication administered in a single dose that is affordable and accessible to the local population if the trial shows treatment effect [24]. At the same time, the incidence of open tibia fractures is higher in Tanzania than in the USA, which facilitates more rapid trial enrollment and more importantly, highlights the large burden of disease in the region. Finally, research partnerships can build research capacity in lower-resource settings for future independent investigations [24].

In conclusion, this is a large, randomized, placebo control trial being conducted at a single center in Dar es Salaam, Tanzania, evaluating the use the local antibiotic gentamicin vs placebo (saline) for the prevention of fracture-related infection after open tibia fractures. Given the pilot study recently completed at the same center, we anticipate being able to enroll and complete the study over a period of 5 years. This study has the potential to provide definitive evidence for the use of local antibiotics as a preventative measure in open tibial fractures, a major source of musculoskeletal morbidity across the globe.

## Trial status

Protocol version 3.0, 01–27-2022. Recruitment began on 1 September 2022. With an estimated 12.5 eligible patients enrolled per month, the target completion date is April 2028.

## Data Availability

The results of the trial will be presented in international peer-reviewed journals. Data will be maintained by the coordinating center, UCSF. Any study data required for future studies may be made available upon reasonable request.

## Abbreviations

FRI(s): Fracture related infection(s)
AE: Adverse event
DSMC: Data and safety monitoring committee
DSMP: Data safety and monitoring plan
NIAMS: National Institute of Arthritis and Musculoskeletal and Skin Diseases
SAE: Serious adverse event
UCSF: University of California, San Francisco
EQ-5D: EuroQol-5 Dimensions, 3-level questionnaire
HIC: High-income countries
LMICs: Low- and middle-income countries
MOI: Muhimbili Orthopaedic Institute
REDCap: Research Electronic Data Capture
